# A New Approach to the Development of Hollow Fiber Membrane Modules for Water Treatment: Mixed Polymer Matrices

**DOI:** 10.3390/membranes13070613

**Published:** 2023-06-21

**Authors:** Dionísio da Silva Biron, Jonathan Cawettiere Espíndola, Eduardo Lucas Subtil, José Carlos Mierzwa

**Affiliations:** 1 International Reference Center on Water Reuse of the University of São Paulo (IRCWR-USP), Environmental and Hydraulic Department (PHA), Polytechnic School of the University of São Paulo (USP), São Paulo 05508-020, SP, Brazil; mierzwa@usp.br; 2 Laboratório de Tecnologias de Tratamento de Águas Urbanas Servidas e Reúso de Água (LabTAUS) of the Federal University of ABC, L005-Block L-Campus Santo André, São Paulo 09210-580, SP, Brazil; eduardo.subtil@ufabc.edu.br

**Keywords:** hollow fiber, membrane technology, PES, PVDF, composite membranes

## Abstract

In this study, mixed matrix hollow fiber polymeric membranes were prepared using polyethersulfone (PES) and polyvinylidene fluoride (PVDF) as polymers in their composition. N-methyl-2-pyrrolidone (NMP) was used as a solvent and demineralized water with an electrical conductivity below 3 μS·cm^−1^ was used as a non-solvent. A new approach to producing enhanced polymeric hollow fiber membranes based on the preparation of a simple blend PVDF/PES solution, and on the conformation of the composite membranes through the extrusion technique followed by the phase inversion process in a non-solvent bath, was applied. The investigation focused on the preparation of polymeric membranes with different polymer ratios and further assessment of the effects of these proportions on the membrane performance and in specific physical properties. The amount of PVDF ranged from 10 to 90% with 10% steps. The presence of PVDF, although it increased the membranes’ plasticity, had a negative effect on the overall mechanical properties of the composite membranes. Scanning electron microscopy (SEM) results showed good dispersion of both polymers in the polymeric matrix. Furthermore, the membrane permeability showed a slight negative correlation with contact angle, suggesting that membrane hydrophilicity played an important role in membrane permeability. Finally, it was found that membranes with low ratios of PVDF/PES may have potential for water treatment applications, due to the combined advantageous properties of PES and PVDF.

## 1. Introduction

In recent decades, global demand for potable water has considerably increased due to population growth. Nevertheless, a large portion of water bodies worldwide that provide water to public drinking water supplies suffers from anthropic- pollution-related problems. According to a recent report from the World Health Organization (WHO), in 2020, 2 billion people lacked safely managed services of drinking water [1]. Therefore, the implementation of wiser water management plans to cope with the ongoing water crisis is of the utmost importance.

In this context, the development of effective treatment technologies capable of producing safe water from different water sources, and in sufficient quantity to supply the population, is a relevant approach to deal with this scenario. Among water treatment processes, membrane separation processes (MSPs) are considered quite competitive considering their potential to carry out the efficient and selective transport of specific substances, replace conventional treatment processes, and enhance the performance of several others [2]. In addition, MSPs are flexible and compatible, in most cases, with other treatment technologies [3].

However, despite the fact that MSPs are considered a mature treatment technology, presenting several technical, economic and environmental advantages, their wide application is still limited considering the problems related to the membrane performance loss. This phenomenon occurs due to membrane fouling, caused by the organic matter contained in the raw water [4] and/or microorganisms (biofouling) [5] that adhere to the microporous wall or inside the pores, and scaling [6] due to the presence of low-solubility salts, such as calcium carbonate and calcium phosphate, among others.

To overcome these limitations in MSPs, typical membrane cleaning procedures are commonly applied for permeate flow recovery. Membrane cleaning involves the use of chemical cleaning at regular times, aeration processes, high tangential velocities along the membrane and/or by backwashing or backpulsing, for instance [7]. On the other hand, alternatives are also being developed to reduce or avoid the occurrence of these phenomena, such as modifying the polymeric matrix or the surface of the membranes. These techniques aim, in general, to increase the membrane’s hydrophilicity, which may result in the reduction of the interaction of fouling compounds with the polymeric matrix [8]. Studies have shown that the presence of nanoparticles of Ag, Cu, Au, ZnO, CuO, TiO_2_ and others within the membranes’ polymeric matrix can provide excellent improvements in their performance due to the antimicrobial capacity of these materials, which consequently prevents the formation of biofouling [9,10]. Another approach is the use of a mixture of two or more polymers, which can provide not only an improvement in their structural strength but also an improvement in their hydrophilicity [11]. For instance, Subtil et al. showed that incorporating 2 wt% polyaniline to PES membranes resulted in a smoother, more hydrophilic membrane with substantial enhancements in permeability and antifouling properties [12].

Despite PES and PVDF polymers being widely disseminated in the literature as microfiltration and ultrafiltration membranes, the combination of these two polymers, aiming to improve their properties, is still sparsely studied. In the studies presented by Madaeni and Pourghorbani (2013) and Salem et al. (2019), the authors demonstrate that these polymers combined in a blend can be efficient in several applications [13,14]. Therefore, the main objective of this work was to evaluate a new approach for developing enhanced polymeric hollow fiber membranes. The method was based on the preparation of a simple blend PVDF/PES solution and on the conformation of the composite membranes through the extrusion technique followed by the phase inversion process in a non-solvent bath. First, 10 types of membranes with different polymer ratios were produced. Secondly, the membranes were characterized using different analysis techniques, such as scanning electron microscopy (SEM) imaging, static water contact angle and mechanical strength measurements. In addition, experimental assays were conducted in a bench-scale membrane reactor in order to determine the porosity and permeability of the membranes. Finally, the gathered data were evaluated and a method to produce an enhanced polymeric membrane was proposed.

## 2. Materials and Methods

### 2.1. Materials

Polyvinylidene fluoride (PVDF) KYNAR 761 from Arkema Química Ltda. (São Paulo, Brazil) and polyethersulfone (PES) VERADEL 3000P from Solvay Advanced Polymer were used as polymers for the preparation of polymeric solutions. N-Methyl-2-Pyrrolidone (NMP) (>99% C_5_H_9_NO, ρ = 1.03 g cm^−3^ and M_w_ = 99.13 g mol^−1^) was obtained from Labsynth^®^ and used as a solvent. Demineralized water (conductivity < 3 μS cm^−1^) was used as a non-solvent.

### 2.2. Membrane Preparation

Pure PVDF and pure PES, as well as PVDF/PES blend membranes, were prepared, and their characteristics were compared. Initially, the polymer powders were dried for 24 h at 120 °C. Then, polymeric solutions were prepared by dissolving 18 wt.% polymer (PVDF, PES or PVDF/PES blend) in the solvent. The polymer powder was slowly added to a beaker containing the solvent. After that, the polymeric solution was maintained under stirring for 24 h at 150 rpm. Before the fiber extrusion process, the degassing process was carried out in an ultrasound bath for 30 min in order to eliminate the presence of air bubbles trapped in the solution. Table 1 presents the composition of the different synthesized membranes.

All membrane development was carried out at the International Reference Center for Water Reuse—IRCWR at the University of São Paulo. These membranes were obtained with the extrusion technique, followed by the phase inversion process, in a non-solvent bath. The extruder has two concentric orifices, which allowed the simultaneous passage of the polymeric fluid and the non-solvent. The bore needle of the spinneret had a diameter of 1.0 mm, and the polymer outlet had an inner diameter of 2.0 mm and an outer diameter of 2.4 mm. The air gap was of 10 cm. The internal fluid (water) flow rate was 30 mL·min^−1^, while the polymer flow rate was 5 mL·min^−1^.

After extrusion, the formed hollow fiber membranes were kept submerged in water for 24 h. Subsequently, the membranes were placed in a solution of isopropyl alcohol and glycerin (70 and 30% *v*/*v*, respectively) for 24 h. After that, the membranes were dried for 48 h before characterization.

### 2.3. Membrane Characterization

#### 2.3.1. Membrane Mechanical Resistance

The mechanical resistance of hollow fiber membranes was evaluated by tensile tests. For that, an Instron universal testing machine (Shimadzu Universal Testing Machine EZ-LX, Kyoto, Japan) with maximum capacity of 5 kN was used. The method applied can be consulted elsewhere [15]. Ten dried samples, with 100 mm length, of each composite membrane were evaluated. Membrane tensile strength at break, elongation at break and Young’s modulus were calculated according to the average value obtained experimentally.

#### 2.3.2. Membranes Morphology

The morphological analysis of the membranes was performed by scanning electron microscopy (SEM) coupled with energy-dispersive X-ray (EDS) analysis, using a Thermo Fisher scanning electron microscope (Waltham, MA, USA), model Quanta 650 FEG. Energy dispersion X-ray spectroscopy (EDS) analyses were performed using Bruker detectors (Billerica, MA, USA), models XFlash 4030 and XFlash 6/60 and Esprit software version 2.3. Membrane samples were immersed into liquid nitrogen and then broken, thus maintaining the membrane’s porous structure integrity. The samples received a carbon coating using a Bal-tec coating system, model SCD-050, with a wire-shaped source. To measure the thickness of the membranes in the SEM images, the Image J software, version 1.54d, was used.

#### 2.3.3. Membranes Hydrophilicity

The hydrophilicity of the synthesized membranes was estimated by the sessile drop technique using a Kino goniometer, model SL150. To carry out this analysis, hollow fiber membranes were cut longitudinally to obtain flat samples. Samples preparation and their respective contact angle measurements were carried out following the ISO 15989 Standard Procedure [16]. For each composite membrane, 3 samples were analyzed. At least 10 measurements of the contact angle of each sample were performed and the mean values were taken.

#### 2.3.4. Membranes Porosity

Apparent porosity was determined using the Archimedes method adapted by [17] Equation (1) was used to determine the apparent porosity of the obtained hollow fiber membranes.

(1) $$P=\frac{W_{wet}-W_{dry}}{\rho_{water}\times V_{membrane}}$$

where P is the porosity of membrane, $W_{wet}$ is the wet membrane mass (g), $W_{dry}$ is the dry membrane mass (g), $\rho_{water}$ is the specific mass of water (g·cm^−3^) and $V_{membrane}$ is the total volume of the membrane sample (cm^3^).

#### 2.3.5. Membranes Permeability

**Figure 1:**
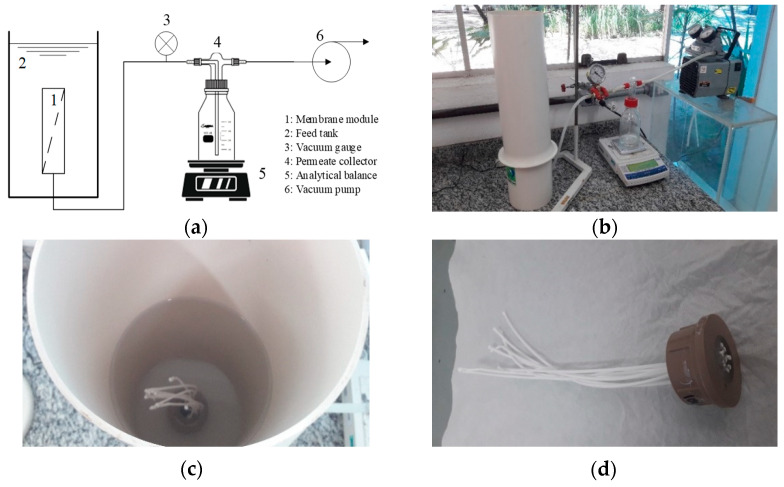
Filtration system used in water permeability tests: (**a**) schematic representation of the MF system, (**b**) photograph of the MF system, (**c**) feed tank containing the membrane module and (**d**) hollow fiber membrane module.

Figure 1 shows the bench scale system in which the permeability tests were carried out for each membrane. The membranes were characterized by the flow of pure water at negative pressures of 5, 10 and 15 kPa and calculated according to Equation (2). The permeate mass was measured on a Shimadzu analytical balance, model UX620H, every 5 min.

(2) $$J_{w}=\frac{V}{A\times \Delta t}$$

where $J_{w}$ is the water flow (L·m^−2^·h^−1^), V is the permeate volume (L), A is the membrane area (m^2^) and Δt is the permeation process time (h).

## 3. Results and Discussion

### 3.1. Mechanical Resistance

**Figure 2:**
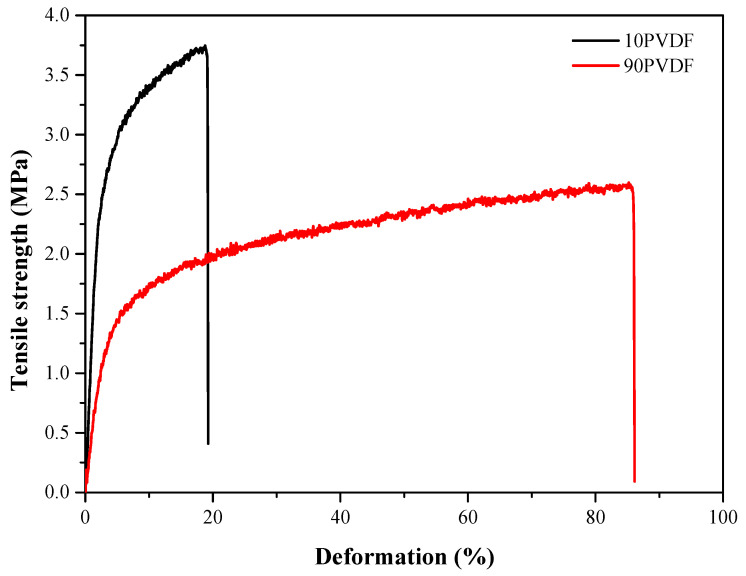
Profile of tensile strength vs. membrane deformation, 10PVDF and 90PVDF.

The mechanical properties of the produced membranes were evaluated by several metrics, such as elongation, tensile strength, yield strength and elasticity [18,19]. Table 2 presents the results of the mechanical resistance tests presented by the synthesized hollow fiber membranes. Figure 2 presents the profile of applied tensile strength in relation to deformation of 10PVDF and 90PVDF hollow fiber membranes.

As can be seen in Table 2, the polymer ratio (PVDF/PES) significantly affected the mechanical properties of the synthesized membranes. Tensile strength at break tended to decrease with the presence of PVDF; however, there was an increase in plasticity of the material, which is indicated by the high elongation at break, a characteristic caused by the presence of PVDF [20]. Another important mechanical property is the Young’s modulus (elasticity of the material), which indicates the maximum force applied to the material without irreversibly deforming it, altering the porous microstructure of the material. The elasticity of the material drastically decreased with the increase of PVDF content, reaching loss values of 86%, from 109.9 MPa for pure PES to 14.9 MPa when 80% PVDF was added. Although the presence of PVDF on the membrane matrix may lead to disadvantages in the mechanical properties of the produced membranes, its presence in low proportions can provide greater ductility to the material. This characteristic would ensure greater mobility in a self-cleaning system that would involve movement or vibration of the fibers. For example, in a MBR system, air, biogas-sparging or backwash and relaxation are commonly applied as membrane cleaning processes [21].

### 3.2. Hydrophilicity

**Figure 3:**
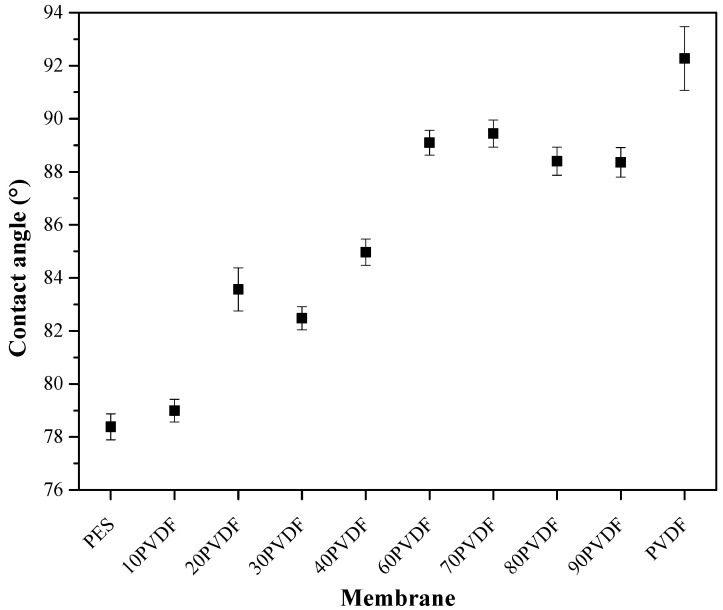
Values of contact angle of the synthesized membranes.

The hydrophilicity of the synthesized membranes was evaluated with water contact angle measurements. Figure 3 presents the results of the mean contact angle values obtained for each membrane. The values of the contact angle measured for the control membranes, 78.4° for the PES membrane and 92.3° for the PVDF membrane, are in agreement with the ones found for hollow fiber membranes in the literature [22,23,24,25].

A trend towards an increase in the value of the contact angle of the membranes by increasing the percentage of PVDF in their composition was observed. This phenomenon was expected due to the more hydrophobic profile of PVDF when compared to the PES. However, this increase was not linear. After a rate of 60% of PVDF in the membrane composition, the contact angle value almost stabilized. This fact can be attributed to the distribution of polymeric particles in the membrane matrix.

### 3.3. Membrane Morphology and Composition

**Figure 4:**
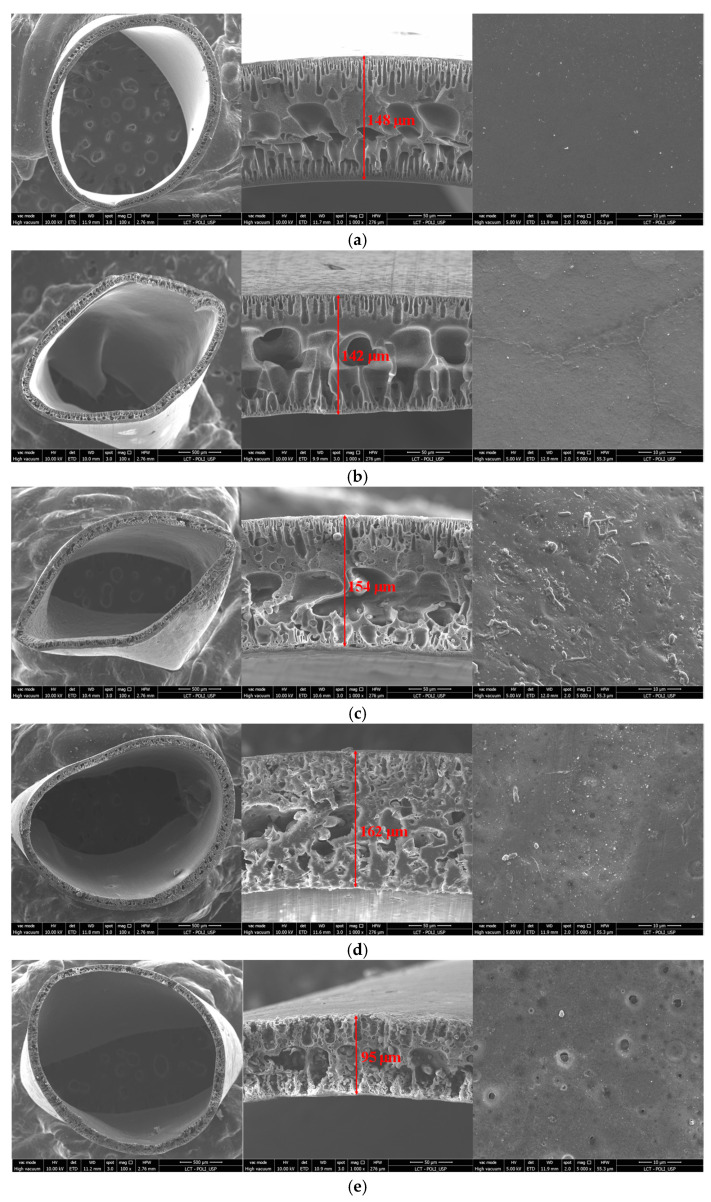
SEM images of morphologies, cross-sections and surface sections of the produced hollow-fiber membranes: (**a**) PES, (**b**) PVDF, (**c**) 10PVDF, (**d**) 20PVDF and (**e**) 30PVDF.

**Figure 5:**
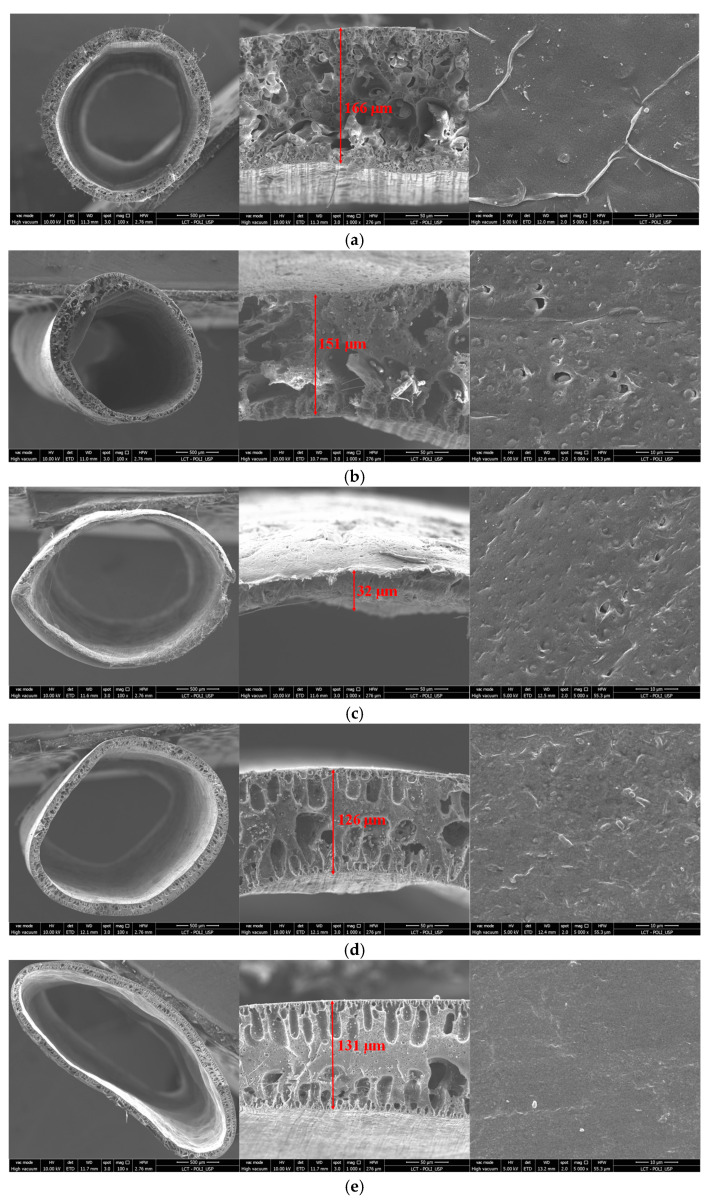
SEM images of morphologies, cross-sections and surface sections of the produced hollow fiber membranes: (**a**) 40PVDF, (**b**) 60PVDF, (**c**) 70PVDF, (**d**) 80PVDF and (**e**) 90PVDF.

All the produced membranes were analyzed with SEM/EDS to evaluate the influence of the polymer concentration ratio on their morphology, structure and chemical composition. Figure 4 and Figure 5 exhibit the surface and cross-sectional SEM images obtained for the PES and PVDF control samples, as well as for the composite membranes.

As can be seen in Figure 4, both control membranes presented an outside dense skin and a double-layered finger-like structure pore. This structure was expected, as it was previously reported by several authors. Large finger-like structures are commonly formed when the coagulation process is fast, while porous sponge-like structures result from slow coagulation rate processes [26,27]. By adding PVDF to the PES polymer matrix, the finger-like structure gradually became deformed and the presence of some voids can be observed. Indeed, when using 70% of PVDF on the polymeric composition, 70PVDF, the membrane presented a structure similar to a sponge-like structure with some voids (Figure 5). Nevertheless, when using a majority percentage of PVDF on the membrane matrix, membranes presenting finger-like structures were again obtained. This fact may have resulted from the immiscibility between the two polymers observed during the mixing step as well as during the extrusion of the hollow fibers, slowing the coagulation process. Thus, when using a polymeric matrix with a polymer ratio (PVDF/PES) close to 1, sponge-like porous structures were shaped due to the delayed demixing process during phase inversion.

By assessing the SEM images of the composite membrane cross-sections, it was possible to determine the average diameter and thickness of the hollow fibers. The results are presented in Table 3.

The determined diameters and thickness of the produced membranes were approximately 2.0 and 0.13 mm, respectively, which is in accordance with the spinneret dimensions. Out of the 60PVDF membrane, there was no statistically significant difference among the determined diameters and thickness of the membrane’s samples. In fact, both membrane samples, 40PVDF and 60PVDF, had smaller diameters than expected. This could again be associated with the immiscibility between the two polymers, leading to disturbances during the configuration of the hollow fiber membrane. Furthermore, visual observations during the extrusion and membrane conformation processes demonstrate the disadvantages of using PVDF/PES ratios above 1.

**Figure 6:**
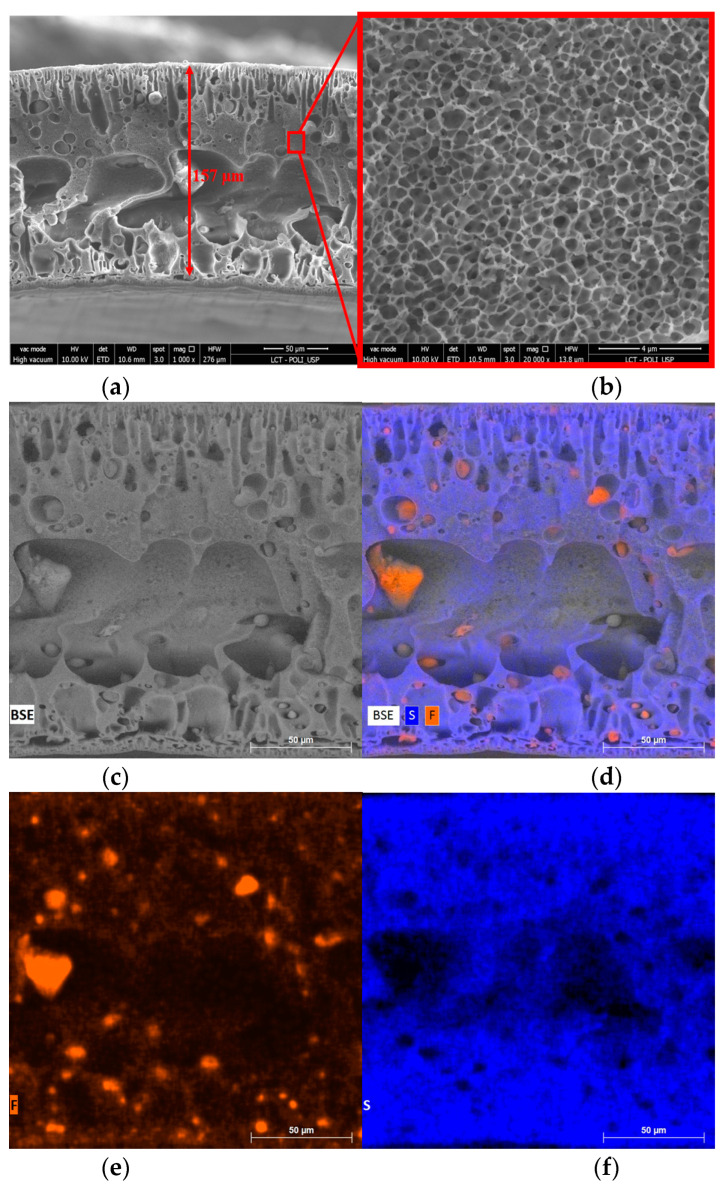
Morphology and composition of the cross-section of the 10PVDF membrane: (**a**) 1 kx, (**b**) 20 kx, (**c**) membrane area for EDS mapping, (**d**) EDS mapping highlighting fluorine and sulfur distribution, (**e**) fluorine distribution in the mixed polymeric matrix and (**f**) sulfur distribution in the mixed polymeric matrix.

**Figure 7:**
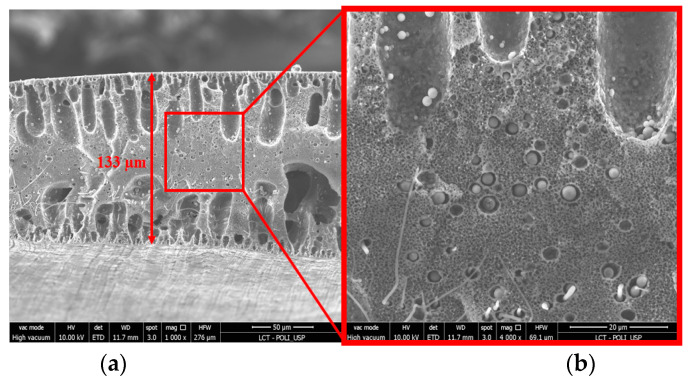

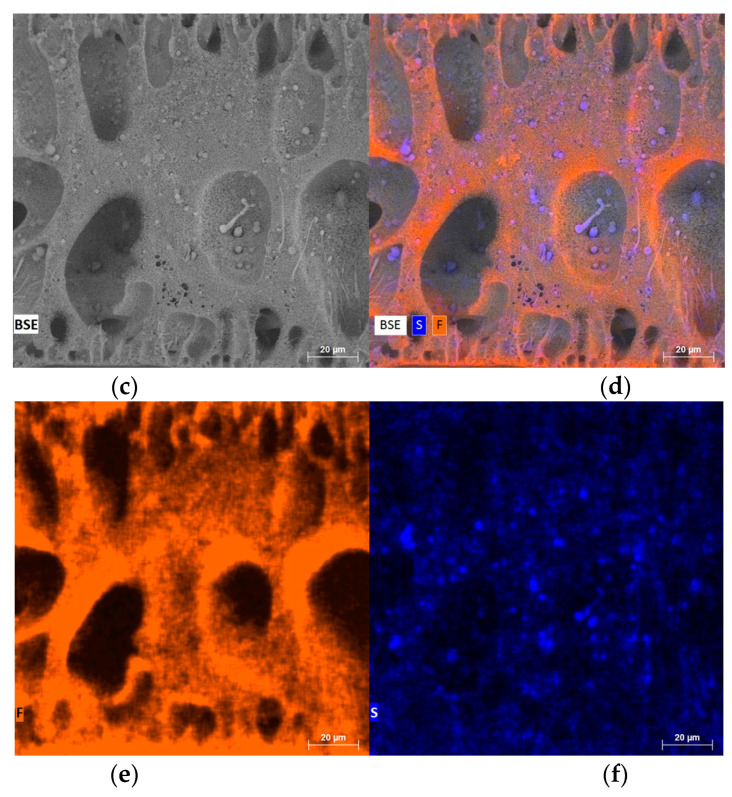
Morphology and composition of the cross-section of the 90PVDF membrane: (**a**) 1 kx, (**b**) 20 kx, (**c**) membrane area for EDS mapping, (**d**) EDS mapping highlighting fluorine and sulfur distribution, (**e**) fluorine distribution in the mixed polymeric matrix and (**f**) sulfur distribution in the mixed polymeric matrix.

Another important aspect observed was the dispersion of the polymers within the membrane matrix. Figure 6 and Figure 7 show the morphology and composition mapping of 10PFDV and 90PVDF, respectively. It is worth mentioning that S (sulfur) was present in the PES polymeric chain while F (fluorine) was present in the PVDF one.

When using mixed polymeric matrices with a higher proportion of PES, it was possible to observe conglomerates of PVDF within the membrane pores (Figure 6), and vice versa (Figure 7). This behavior is also related to the immiscibility between the two polymers and the different coagulation times, leading to the migration of some of the polymer into the membrane pores.

### 3.4. Permeability and Porosity

**Figure 8:**
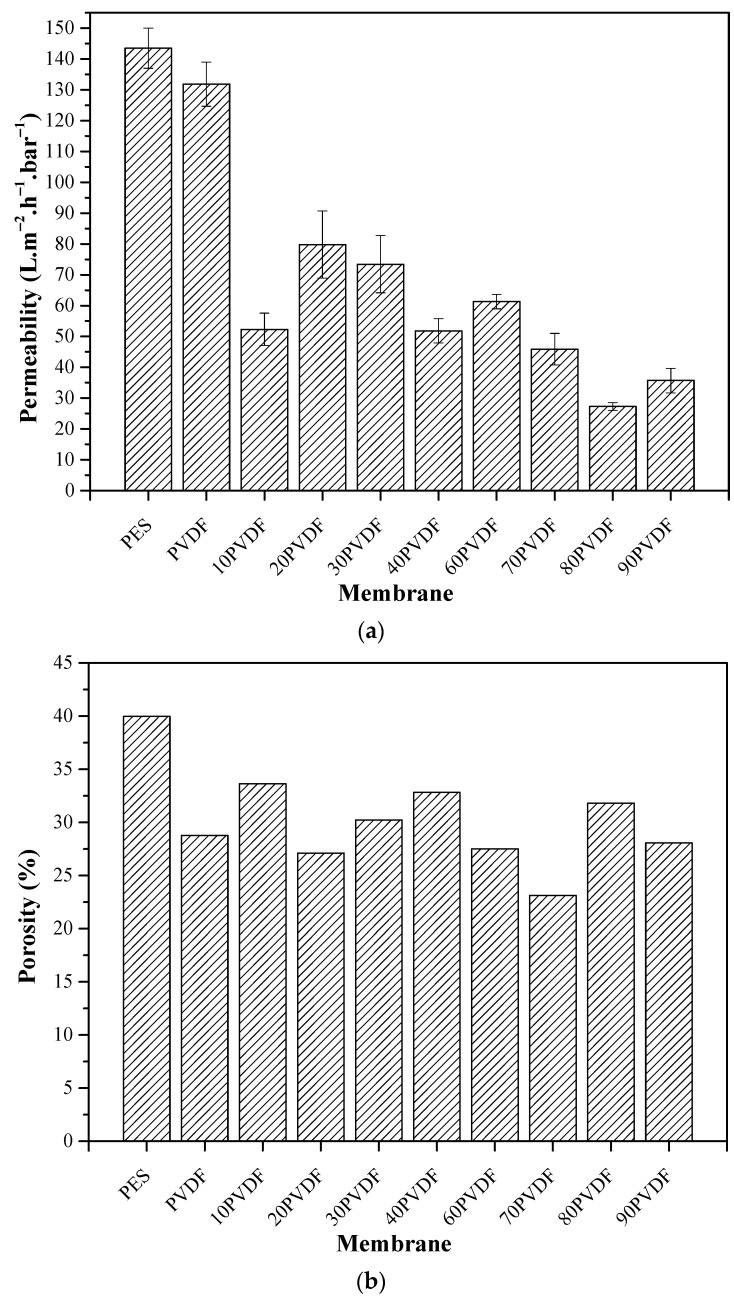
Permeability of hollow fiber membranes with demineralized water (**a**) and apparent porosity (**b**).

Figure 8 shows the water permeability and porosity of the membranes produced in this study. As can be seen, the addition of PVDF to the PES polymeric matrix resulted in a considerable reduction in hydraulic permeability. Pure PES and PVDF membranes provided higher values of water permeability, which favors their efficiency in producing greater permeate volumes. However, as observed by the mechanical strength analysis, membranes with PVDF in their structure presented greater deformation at rupture (Table 2), which may be an important characteristic for a process that involves shearing of the fibers.

Pure PES and PVDF membranes showed permeability values of 144 and 132 L·m^−2^·h^−1^·bar^−1^, respectively. When mixing the two polymers, a considerable decrease in permeability values was obtained, reaching reduction values of up to 80% (in the case of the 80PVDF membrane). However, these values are still compatible with the ones found in the literature [28,29,30]. In a study presented by Alsalhy et al. [28], the authors reached water permeability values between 19.2 and 236.4 L·m^−2^·h^−1^·bar^−1^. In another study, Chu et al. [29] obtained permeate fluxes between 7.3 and 254.6 L·m^−2^·h^−1^·bar^−1^, but at a transmembrane pressure of 600 kPa.

Changes in the composition of the membranes, in addition to causing variations in their mechanical and hydrophilic characteristics, also altered their permeability. Membrane permeability showed a slight negative correlation with contact angle (Figure 3 and Figure 8a), suggesting that increased membrane hydrophilicity may be responsible for increased permeability. By increasing the concentration of PVDF, a tendency in decreasing permeability was observed.

The decrease in the permeate flux cannot only be attributed to the physicochemical characteristics of the different polymers, but also to their distribution in the mixed-matrix structure. As can be seen in the SEM images (Figure 7), the polymers presented good dispersion; however, some pores were filled by the polymer present in lower concentration. A similar behavior was also observed in the studies presented by Madaeni and Pourghorbani (2011) and Salem et al. (2019) [13,14]. This phenomenon, which occurred during phase inversion, may have reduced the pore availability, causing loss of permeate flux. Another important feature is the phase inversion time, which occurred differently for each tested polymer [7]. In addition, composite membranes containing lower amounts of PVDF in their structure showed larger finger-shaped pores. This characteristic may be associated with the greater permeability observed for these membranes, since there is a greater void for the passage of fluid through the hollow fiber wall.

Nevertheless, membranes prepared with greater amounts of PVDF in their structure demonstrated a higher probability of collapsing during permeability tests. The collapse of the fiber walls occurs due to the negative pressure generated inside the tested membranes. This phenomenon is due to the greater ductility (i.e., the degree of deformation that a material resists until the moment of its fracture) of PVDF compared to PES. Indeed, according to the mechanical characterization data obtained (Table 2), it is possible to verify the higher ductility of membranes containing higher proportions of PVDF.

Additionally, as can be seen in Figure 8b, the porosity of pure PES and PVDF membranes was 40 and 29%, respectively. When PVDF was added to the polymer matrix, the porosity of the composite membranes decreased to 17%. However, the porosity of the different samples remained stable even after increasing the amount of PVDF in the polymeric matrix. It is widely known that the spinning process has an important effect on the membrane pore structure. Despite the spinning conditions being the same, there were still some differences in the spinning process when using the PES/PVDF mixture. For the PES membrane, since it was not introduced to PVDF, when the membrane was taken up during the spinning process, the elongation deformation occurred mainly from the part extruding from the spinneret to the part that was not completely solidified due to the pull force, while in the spinning process of the other membranes, it was mainly the introduction of the PVDF that bore the pull force, so the separation layer that extruded from the spinneret to the incompletely solidified part did not produce elongation deformation as large as PES. This phenomenal can be explained by the greater viscosity of PVDF in relation to PES [31,32,33].

## 4. Conclusions

Membrane composition modification is a promising approach to obtaining membranes with desirable properties for separation process. In this study, the effectiveness of combining two different polymers, PVDF and PES, was assessed in terms of the morphological properties and performance of hollow fiber composite membranes. Significant changes in the membranes’ properties were observed by varying the polymers’ ratio. In general, the mechanical properties of the composite membranes were inferior to those of pure PES; however, the presence of PVDF on the PES polymeric matrix showed an improvement in the membrane plasticity, causing a positive effect on fiber mobility. Membrane morphologies obtained via SEM/EDS showed that the composite membranes present a homogeneously mixed matrix containing both polymers, despite the poor miscibility between PVDF and PES. The hydrophilicity of the membranes decreased by increasing the PVDF content, due to its hydrophobic characteristic. The loss of permeate flux and the reduction in porosity of the composite membranes were also attributed mainly to the hydrophobic characteristics of PVDF.

Finally, it was found that membranes with low ratios of PVDF/PES may be of interest for water treatment applications due to the combined advantageous properties of PES and PVDF. Indeed, currently, our group has been developing a membrane module prototype for a membrane bioreactor for wastewater treatment applications based on the obtained results. The findings of this work, in addition to promoting a more comprehensive understanding of the effect of mixed polymeric matrices on membrane performance, may also motivate future investigations in this field of exponential growth, i.e., membrane technology for water treatment, including for reuse purposes.

## Figures and Tables

**Table 1:** Composition, in terms of polymer proportion, of the synthesized membranes.

Membrane	PVDF (%)	PES (%)
PES	0	100
PVDF	100	0
10PVDF	10	90
20PVDF	20	80
30PVDF	30	70
40PVDF	40	60
60PVDF	60	40
70PVDF	70	30
80PVDF	80	20
90PVDF	90	10

**Table 2:** Result of the mechanical resistance for the different synthesized membranes.

Membrane	Tensile Strength at Break (MPa)	Elongation at Break (%)	Young’s Modulus (MPa)
PES	5.2 ± 0.5	65.6 ± 12.8	109.9 ± 7.9
10PVDF	3.9 ± 0.3	15.4 ± 4.3	105.5 ± 13.6
20PVDF	3.6 ± 0.4	14.1 ± 7.4	78.8 ± 20.3
30PVDF	3.3 ± 0.2	26.4 ± 6.2	69.5 ± 12.4
40PVDF	3.5 ± 0.3	29.7 ± 7.4	82.7 ± 4.4
60PVDF	2.9 ± 0.4	47.6 ± 11.4	44.9 ± 0.2
70PVDF	2.4 ± 0.1	107.7 ± 16.2	32.8 ± 4.7
80PVDF	1.6 ± 0.1	101.8 ± 10.9	14.9 ± 1.9
90PVDF	2.7 ± 0.2	105.6 ± 25.9	33.5 ± 7.2
PVDF	2.3 ± 0.3	176.6 ± 40.3	27.5 ± 5.2

**Table 3:** Physical characteristics of the hollow fiber membranes.

Membrane	Diameter (mm)	Standard Deviation (mm)	Thickness (mm)	Standard Deviation (mm)
PES	2.13	0.10	0.14	0.01
PVDF	2.28	0.30	0.15	0.02
10PVDF	1.98	0.13	0.12	0.01
20PVDF	2.00	0.47	0.14	0.01
30PVDF	2.14	0.03	0.14	0.01
40PVDF	1.91	0.20	0.15	0.03
60PVDF	1.65	0.11	0.11	0.01
70PVDF	2.29	0.18	0.13	0.04
80PVDF	2.14	0.13	0.14	0.01
90PVDF	2.08	0.21	0.12	0.02

## Data Availability

The data presented in this study are available upon request from the corresponding author.
